# Supplementary material for: Inhibiting heme piracy by pathogenic Escherichia coli using de novo-designed proteins
Source: Nat Commun. 2025 Jul 9;16:6066. doi: 10.1038/s41467-025-60612-9 (PMC12241658; doi:10.1038/s41467-025-60612-9)
Supplement: Supplementary file 1 — Supplementary Information [file 41467_2025_60612_MOESM1_ESM.pdf]

Supplementary Information for:

Inhibiting heme piracy by pathogenic *Escherichia coli* using *de novo*-designed proteins

Daniel R. Fox[1,2,3], Kazem Asadollahi[3], Imogen Samuels[3], Bradley A. Spicer[4], Ashleigh Kropp[1,2], Christopher J. Lupton[4], Kevin Lim[5], Chunxiao Wang[3], Hari Venugopal[6], Marija Dramicanin[5,7], Gavin J. Knott[4*], Rhys Grinter[1,2,3*]

[1]Department of Microbiology, Biomedicine Discovery Institute, Monash University, Clayton 3800, Australia

[2]Centre for Electron Microscopy of Membrane Proteins, Monash Institute of Pharmaceutical

Sciences, Parkville, 3052, Victoria, Australia

[3]Department of Biochemistry and Pharmacology, Bio21 Molecular Science and Biotechnology Institute, The University of Melbourne, Parkville, Victoria 3010, Australia

[4]Department of Biochemistry and Molecular Biology, Biomedicine Discovery Institute, Monash University, Clayton 3800, Australia

[5]The Walter and Eliza Hall Institute of Medical Research, Parkville, Victoria 3052, Australia

[6]Ramaciotti Centre for Cryo-Electron Microscopy, Biomedicine Discovery Institute, Monash University, Clayton 3800, Australia

[7]Department of Medical Biology, University of Melbourne, Parkville, Victoria 3010, Australia

* Corresponding authors. Email: rhys.grinter@unimelb.edu.au, gavin.knott@monash.edu.au

Supplementary Figures

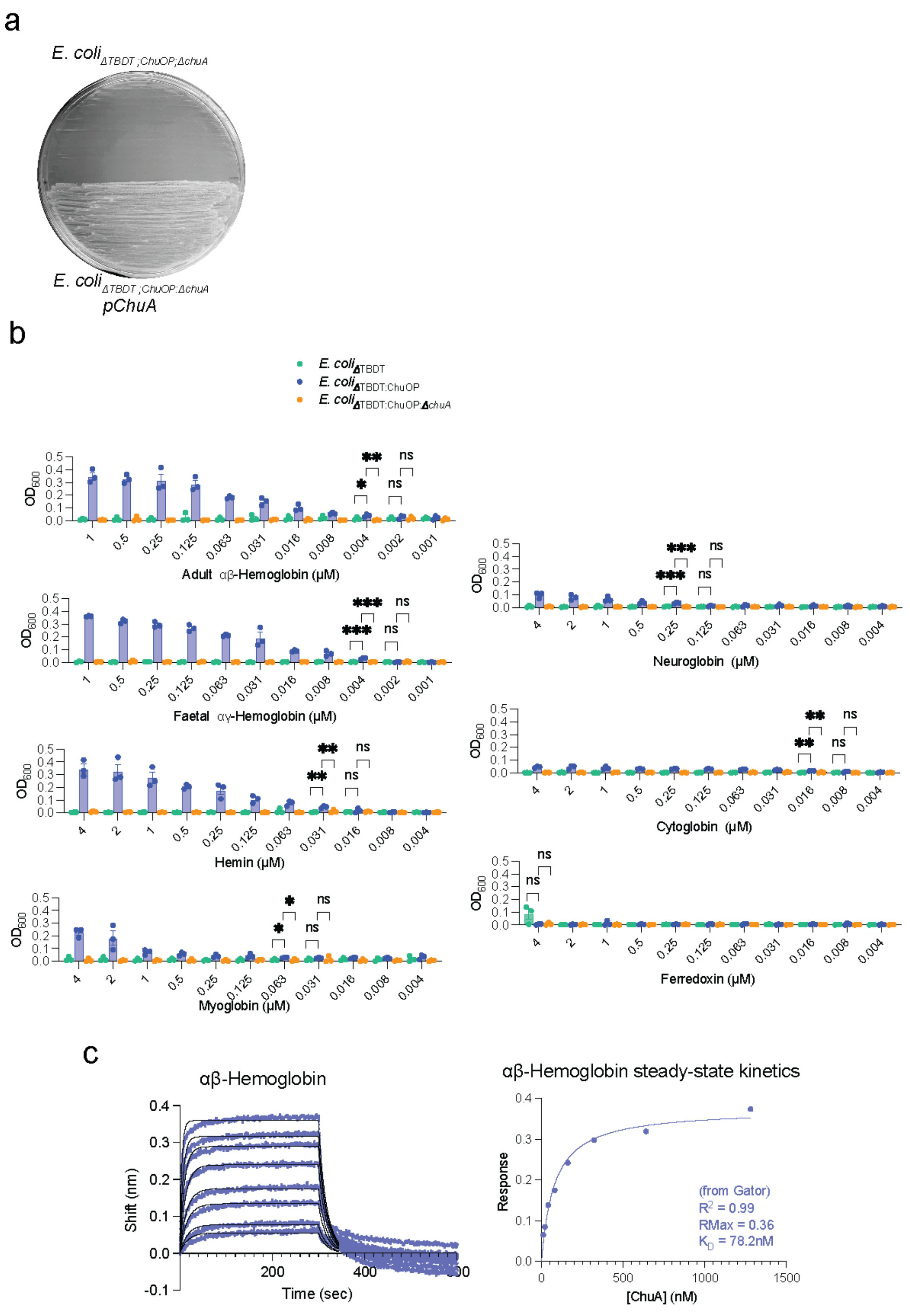

Supplementary Fig. 1: Capacity of ChuA to target different heme-containing substrates. **(a)** A hemoglobin agar plate streaked with *E. coli*$_{\Delta TBDT:ChuOP:\Delta chuA}$ or *E. coli*$_{\Delta TBDT:ChuOP:\Delta chuA}$ pChuA, showing ChuA is required in this strain for hemoglobin-dependent growth. **(b)** Liquid culture growth assays with different heme-containing substrates, with *E. coli*$_{\Delta TBDT}$, *E. coli*$_{\Delta TBDT:ChuOP,}$ or *E. coli*$_{\Delta TBDT:ChuOP:\Delta chuA}$, showing the minimum substrate concentrations able to support Chua-dependent growth. (n=3, biological replicates, statistical significance was determined through a one-way ANOVA with multiple comparisons tests. $P < 0.05$ was considered statistically significant. **(c)** Representative BLI sensorgram trace (left) and associated steady-state binding kinetics (right) of αβHb binding to ChuA. BLI experiments was performed twice (n=2), with comparable results. K_D value represents the average of these the two experiments.

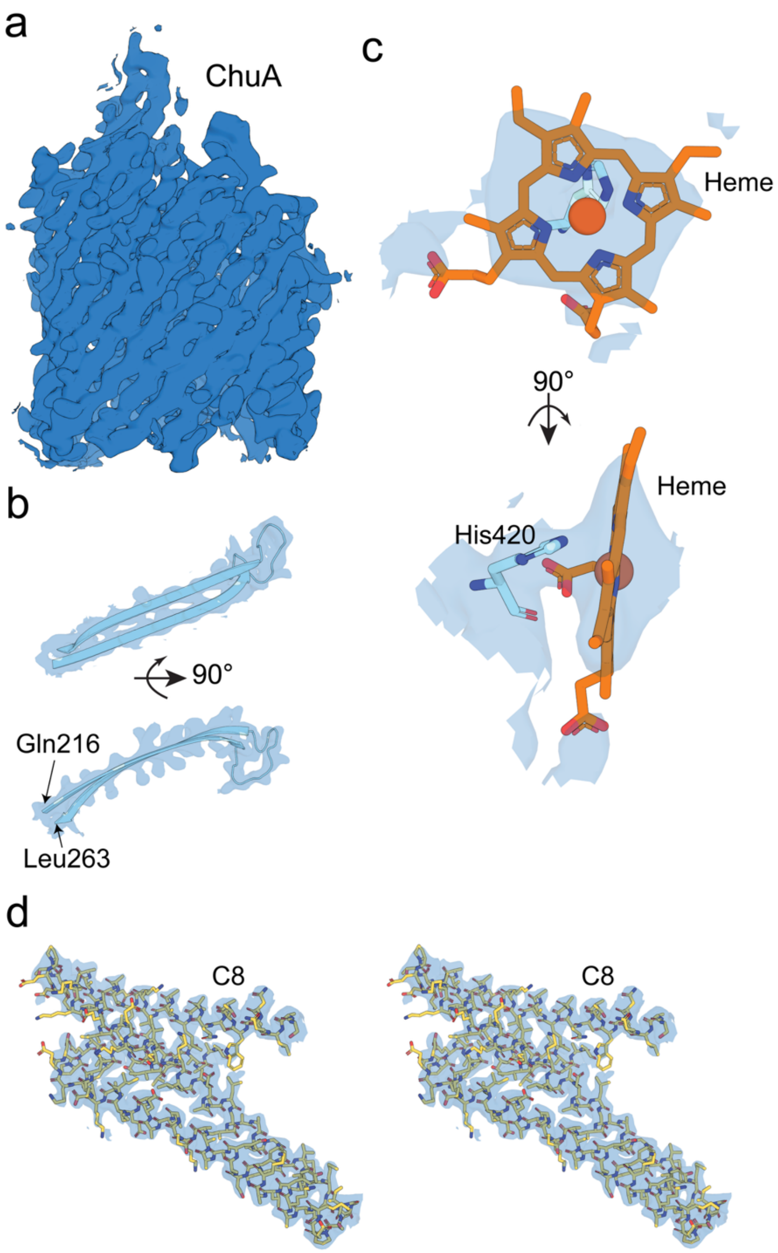

Supplementary Fig. 2: Examples of electron density from the ChuA-heme and C8 binder crystal structures. (a) A 2Fo-Fc composite omit map density map covering the asymmetric unit of the ChuA-heme crystal structure. (b) A zoomed-in view of the ChuA-heme map showing side chain density for two β-sheets. (c) A zoomed-in view of the ChuA-heme map showing density for bound heme. (d) A 2Fo-Fc composite omit map density map covering one C8 molecule in the asymmetric unit of the crystal structure. All maps are contoured to 1 σ.

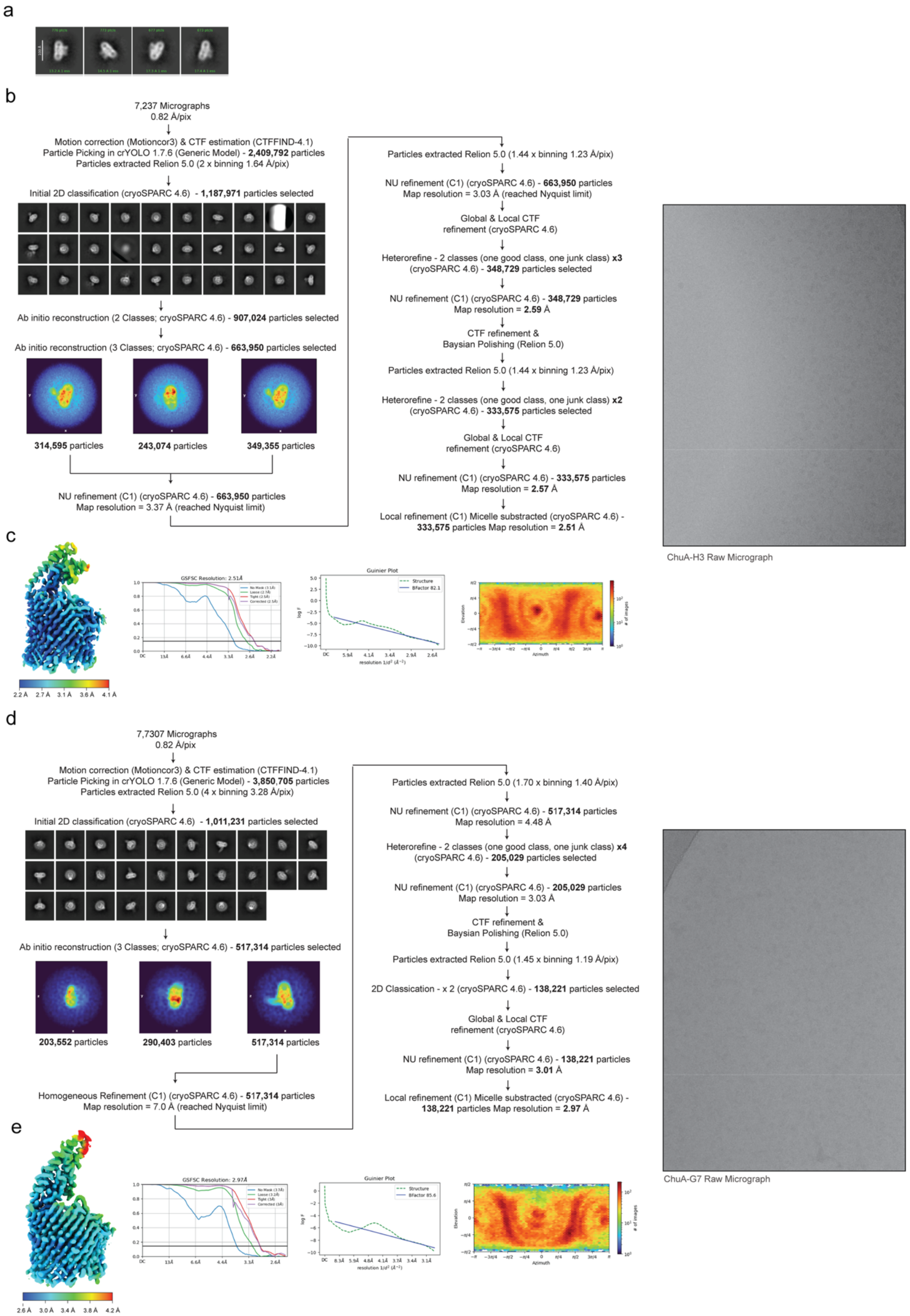
a
b
7,237 Micrographs
0.82 Å/pix
Motion correction (Motioncor3) & CTF estimation (CTFFIND-4.1)
Particle Picking in crYOLO 1.7.6 (Generic Model) - 2,409,792 particles
Particles extracted Relion 5.0 (2 x binning 1.64 Å/pix)
Initial 2D classification (cryoSPARC 4.6) - 1,187,971 particles selected
Ab initio reconstruction (2 Classes; cryoSPARC 4.6) - 907,024 particles selected
Ab initio reconstruction (3 Classes; cryoSPARC 4.6) - 663,950 particles selected
314,595 particles
243,074 particles
349,355 particles
NU refinement (C1) (cryoSPARC 4.6) - 663,950 particles
Map resolution = 3.37 Å (reached Nyquist limit)
Particles extracted Relion 5.0 (1.44 x binning 1.23 Å/pix)
NU refinement (C1) (cryoSPARC 4.6) - 663,950 particles
Map resolution = 3.03 Å (reached Nyquist limit)
Global & Local CTF
refinement (cryoSPARC 4.6)
Heterorefine - 2 classes (one good class, one junk class) x3
(cryoSPARC 4.6) - 348,729 particles selected
NU refinement (C1) (cryoSPARC 4.6) - 348,729 particles
Map resolution = 2.59 Å
CTF refinement &
Baysian Polishing (Relion 5.0)
Particles extracted Relion 5.0 (1.44 x binning 1.23 Å/pix)
Heterorefine - 2 classes (one good class, one junk class) x2
(cryoSPARC 4.6) - 333,575 particles selected
Global & Local CTF
refinement (cryoSPARC 4.6)
NU refinement (C1) (cryoSPARC 4.6) - 333,575 particles
Map resolution = 2.57 Å
Local refinement (C1) Micelle substracted (cryoSPARC 4.6) -
333,575 particles Map resolution = 2.51 Å
ChuA-H3 Raw Micrograph
c
2.2 Å 2.7 Å 3.1 Å 3.6 Å 4.1 Å
GSFSC Resolution: 2.51Å
Guinier Plot
Structure
BFactor 82.1
Azimuth
Elevation
d
7,7307 Micrographs
0.82 Å/pix
Motion correction (Motioncor3) & CTF estimation (CTFFIND-4.1)
Particle Picking in crYOLO 1.7.6 (Generic Model) - 3,850,705 particles
Particles extracted Relion 5.0 (4 x binning 3.28 Å/pix)
Initial 2D classification (cryoSPARC 4.6) - 1,011,231 particles selected
Ab initio reconstruction (3 Classes; cryoSPARC 4.6) - 517,314 particles selected
203,552 particles
290,403 particles
517,314 particles
Homogeneous Refinement (C1) (cryoSPARC 4.6) - 517,314 particles
Map resolution = 7.0 Å (reached Nyquist limit)
Particles extracted Relion 5.0 (1.70 x binning 1.40 Å/pix)
NU refinement (C1) (cryoSPARC 4.6) - 517,314 particles
Map resolution = 4.48 Å
Heterorefine - 2 classes (one good class, one junk class) x4
(cryoSPARC 4.6) - 205,029 particles selected
NU refinement (C1) (cryoSPARC 4.6) - 205,029 particles
Map resolution = 3.03 Å
CTF refinement &
Baysian Polishing (Relion 5.0)
Particles extracted Relion 5.0 (1.45 x binning 1.19 Å/pix)
2D Classication - x 2 (cryoSPARC 4.6) - 138,221 particles selected
Global & Local CTF
refinement (cryoSPARC 4.6)
NU refinement (C1) (cryoSPARC 4.6) - 138,221 particles
Map resolution = 3.01 Å
Local refinement (C1) Micelle substracted (cryoSPARC 4.6) -
138,221 particles Map resolution = 2.97 Å
ChuA-G7 Raw Micrograph
e
2.6 Å 3.0 Å 3.4 Å 3.8 Å 4.2 Å
GSFSC Resolution: 2.97Å
Guinier Plot
Structure
BFactor 85.6
Azimuth
Elevation

Supplementary Fig. 3: CryoEM processing data for ChuA-Hb and binder complexes. **(a)** 2d class averages from grids prepared with a 1:1 ratio of ChuA to αβHb, showing only ChuA-alone class averages were obtained. **(b)** Data processing workflow for the ChuA-H3 binder complex. **(c)** ChuA-H3 binder final map coloured by local resolution, and plots of resolution, B-factor and particle angle distribution. **(d)** Data processing workflow for the ChuA-G7 binder complex. **(e)** ChuA-G7 binder final map coloured by local resolution, and plots of resolution, B-factor and particle angle distribution.

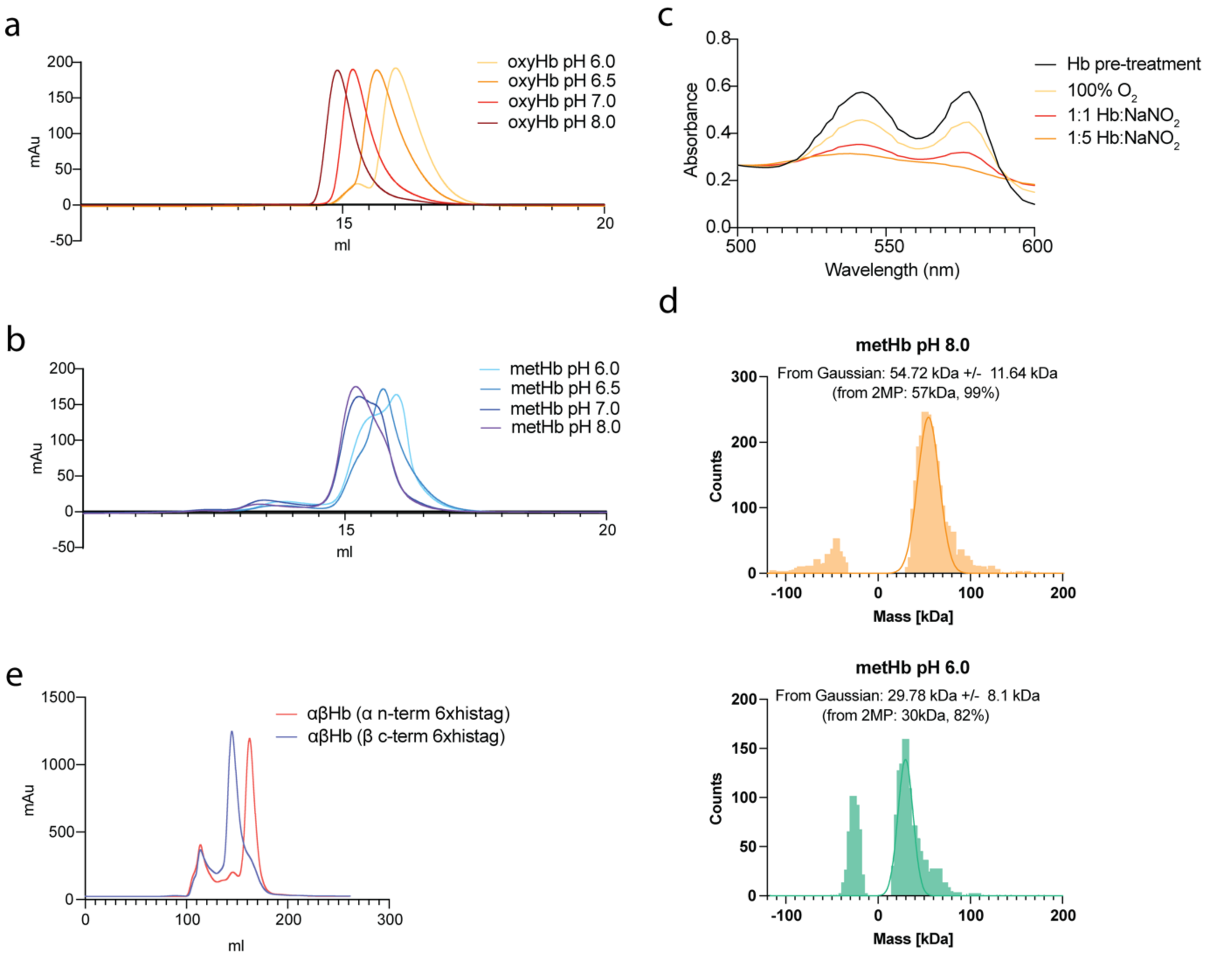

Supplementary Fig. 4: The effect of pH and redox state on hemoglobin oligomeric state. Analytical SEC of oxy-αβHb **(a)** and met-αβHb **(b)**, at pH 6 to 8, showing that αβHb is predominantly tetrameric at pH 8.0, and largely dimeric at pH 6.0. **(c)** The change in absorbance of oxy-αβHb between 500-600 nm, after treatment with O_2 or $NaNO_2$ to generate met-αβHb. **(d)** Mass-photometry data confirming that met-αβHb is predominantly tetrameric at pH 8.0, and largely dimeric at pH 6.0. **(e)** SEC profile of recombinant αβHb pH7.4, 6xhis-tagged at either the n-term of the α-subunit or at the c-term of the β-subunit, showing that tagging the α-subunit induces dimerization.

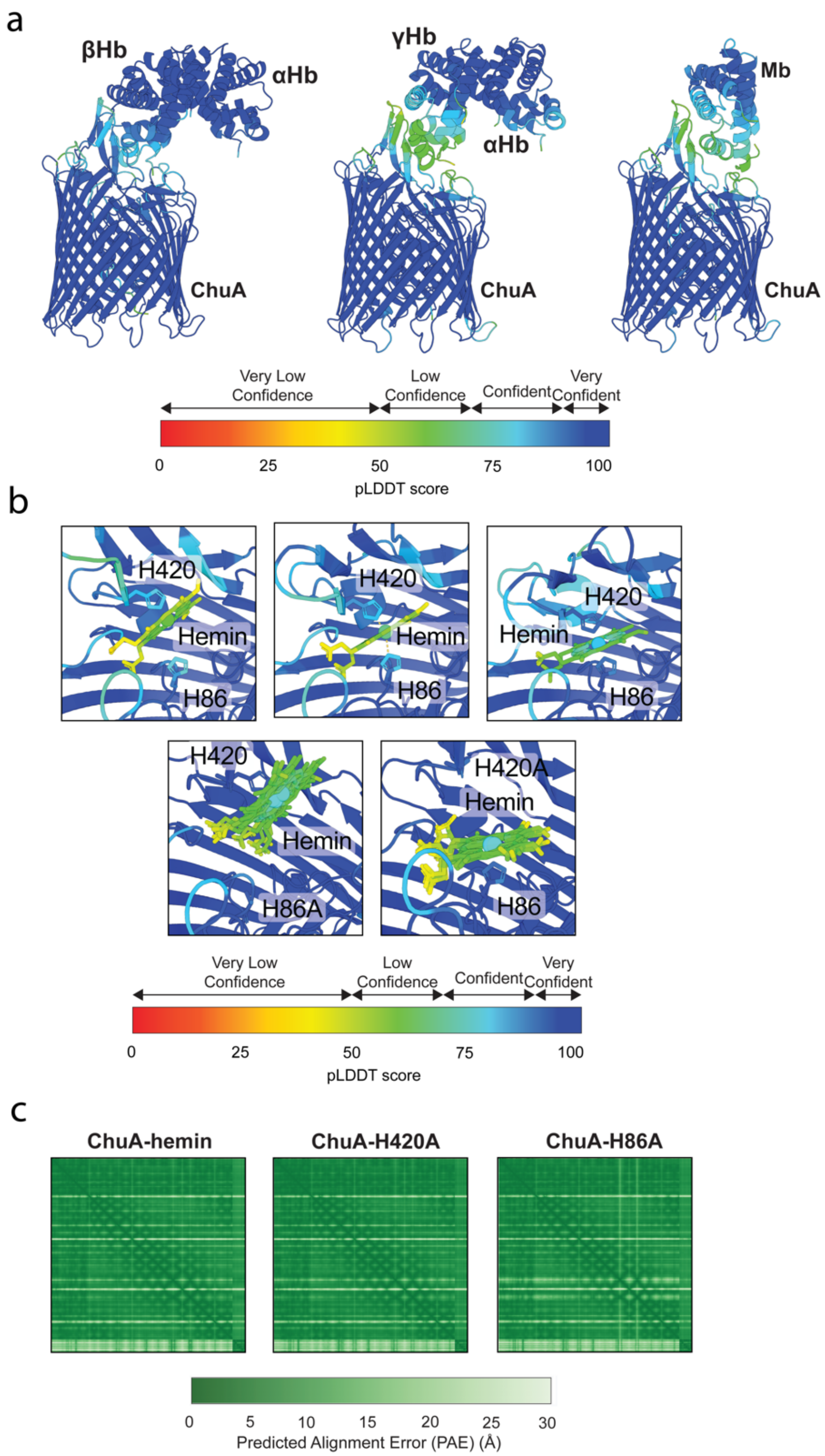
a
βHb
αHb
ChuA
γHb
αHb
ChuA
Mb
ChuA
Very Low Confidence
Low Confidence
Confident
Very Confident
0
25
50
75
100
pLDDT score
b
H420
Hemin
H86
H420
Hemin
H86
H420
Hemin
H86
H420
Hemin
H86A
H420A
Hemin
H86
Very Low Confidence
Low Confidence
Confident
Very Confident
0
25
50
75
100
pLDDT score
c
ChuA-hemin
ChuA-H420A
ChuA-H86A
0
5
10
15
20
25
30
Predicted Alignment Error (PAE) (Å)

Supplementary Fig. 5: AlphaFold modelling quality indicators for ChuA-substrate complexes. **(a)** AlphaFold2 models of the ChuA-αβHb, ChuA-αγHb, and ChuA-Mb complexes coloured by pLDDT confidence score. **(b)** AlphaFold3 models of ChuA-hemin complexes coloured by pLDDT confidence score. **(c)** pAE scores for the AlphaFold3 ChuA-hemin models.

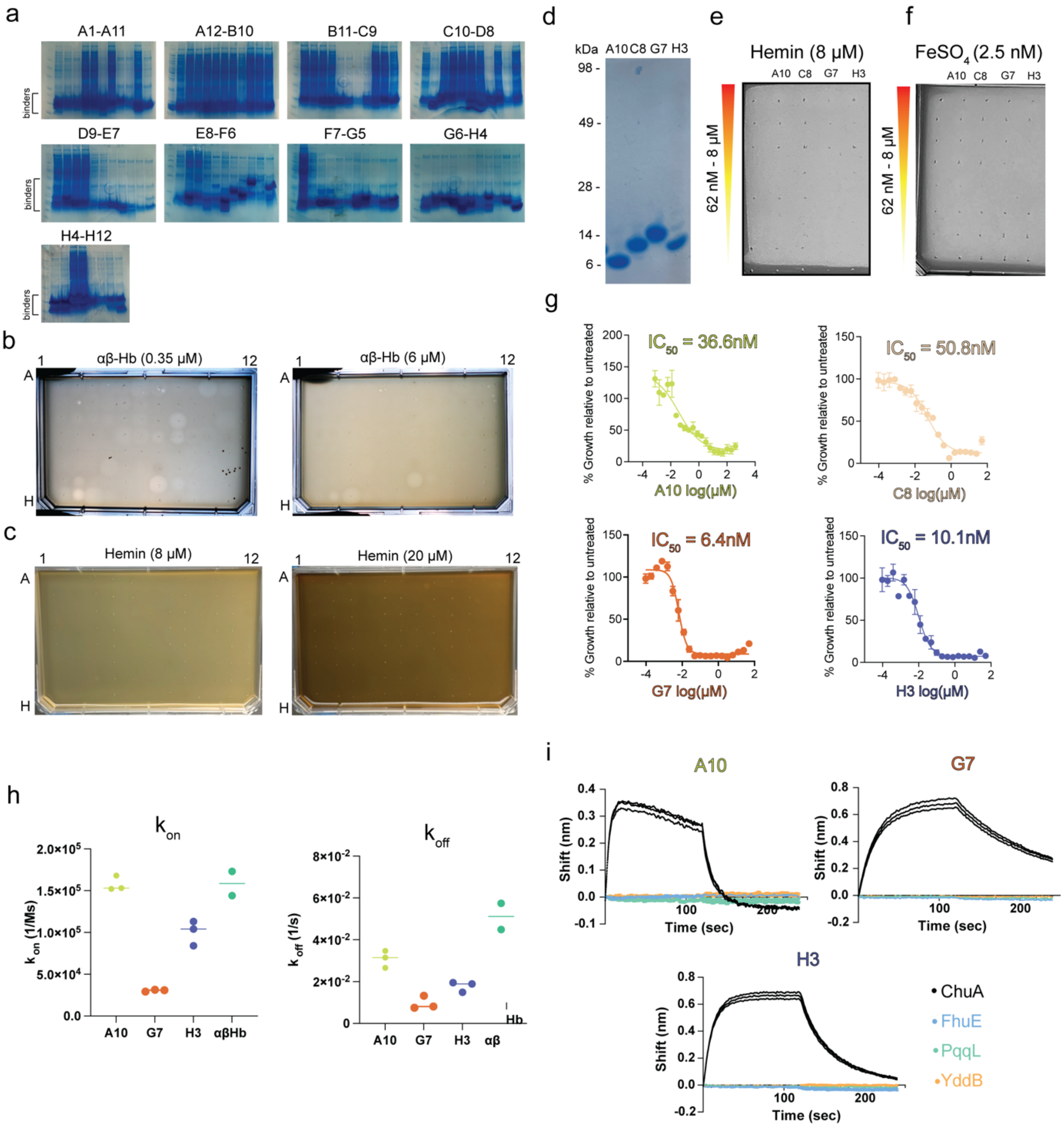

a
A1-A11
A12-B10
B11-C9
C10-D8
D9-E7
E8-F6
F7-G5
G6-H4
H4-H12
binders
b
αβ-Hb (0.35 μM)
αβ-Hb (6 μM)
1
12
A
H
c
Hemin (8 μM)
Hemin (20 μM)
d
kDa
A10 C8 G7 H3
98
49
28
14
6
e
Hemin (8 μM)
62 nM - 8 μM
f
FeSO4 (2.5 nM)
g
IC50 = 36.6nM
IC50 = 50.8nM
IC50 = 6.4nM
IC50 = 10.1nM
% Growth relative to untreated
A10 log(μM)
C8 log(μM)
G7 log(μM)
H3 log(μM)
h
kon
koff
kon (1/Ms)
koff (1/s)
A10
G7
H3
αβHb
i
A10
G7
H3
Shift (nm)
Time (sec)
ChuA
FhuE
PqqL
YddB

Supplementary Fig. 6: Screening and validation of *de novo* designed ChuA binders. (a) SDS-PAGE analysis of initial purification of binders for functional screening. The ability of binder to inhibit an overlay of *E. coli*$_{\Delta TBDT:ChuOP}$ on agar containing different concentrations of αβHb **(b)** or hemin **(c)**. **(d)** SDS-PAGE analysis of large-scale purification of ChuA binders selected for further analysis. **(e)** A soft agar overlay assay of *E. coli*$_{\Delta TBDT:ChuOP}$, grown on iron-limited LB agar containing 8 µM hemin, spotted with 2-fold serial dilution of *de novo* ChuA binders A10, C8, G7 and H3 (8 µM – 62 nM). **(f)** A soft agar overlay assay of *E. coli*$_{\Delta TBDT:ChuOP}$, grown on LB agar containing 2.5 nM $FeSO_4$, spotted with *de novo* as in panel d. **(g)** Binder IC_{50} of *E. coli*$_{\Delta TBDT:ChuOP}$ grown iron limited LB broth containing 0.05 µM αβHb, with 2-fold serially diluted *de novo* binders A10, C8, G7 or H3. IC_{50} values were calculated as a % relative to the growth of the untreated control. Data (n=3) displayed as mean ± s.e.m. **(h)** a plot of k_{on} and k_{off} rates for ChuA to binding proteins measured by BLI. **(i)** Triplicate BLI sensorgram traces (top) of A10, G7 and H3 against ChuA, and control proteins FhuE, YddB and PqqL. Binding response curves were only observed for ChuA. All BLI experiments were performed three times (n=3), with comparable results.

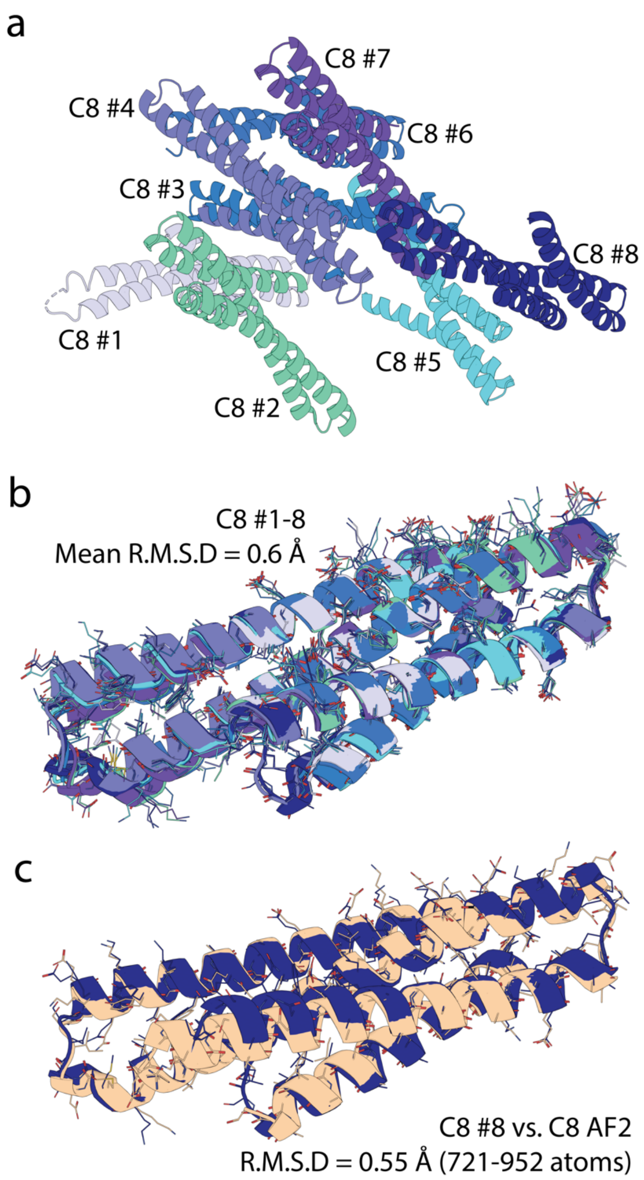

Supplementary Fig. 7: The ChuA binder C8 computational model closely matches the crystal structure. (a) A cartoon representation of the eight C8 molecules in the asymmetric unit of the crystal structure of C8 crystal structure. **(b)** A superimposition of the eight C8 molecules from the C8 crystal structure, showing they exhibit some variation in both sidechain and backbone conformation. **(c)** A superimposition of the C8 AlphaFold2 model (tan) and a representative C8 molecule from the crystal structure (dark blue), show experiment and prediction match closely.

Supplementary Tables

Supplementary Table 1: BLI statistical parameters for globins and binding proteins.

	Steady State Result R2	RMax	KD	Kinetics Summary koff(1/s)	kon(1/Ms)	KD(M)	Full R2	Full X2
Hb dimer rpt 1	0.99	0.36	7.82E-08	0.0574	144000	4.00E-07	0.99	1.681
Hb dimer rpt 2	0.97	0.19	6.48E-08	0.0449	173000	2.59E-07	0.985	0.475
**Hb dimer average**			**7.15E-08**	**0.05115**	**158500**	**3.30E-07**		
A10 rpt 1	1	0.87	9.42E-08	0.0488	168000	2.91E-07	0.997	1.95
A10 rpt 2	1	1.7	1.29E-07	0.0266	205000	1.29E-07	0.997	2.237
A10 rpt 3	1	1.63	1.30E-07	0.0315	153000	2.06E-07	0.999	1.955
A10 rpt 4	1	1.37	1.55E-07	0.0347	152000	2.29E-07	0.998	2.763
**A10 average**			**1.27E-07**	**0.0354**	**169500**	**2.14E-07**		
G7 rpt 1	0.99	1.03	7.84E-08	0.00807	29200	2.76E-07	0.998	2.068
G7 rpt 2	1	1.27	8.58E-08	0.00741	31400	2.36E-07	0.999	2.244
G7 rpt 3	1	1.28	8.44E-08	0.00753	30900	2.44E-07	0.999	1.974
G7 rpt 4	1	0.88	9.10E-08	0.0132	73600	1.79E-07	0.992	1.759
**G7 average**			**8.49E-08**	**0.0090525**	**41275**	**2.34E-07**		
H3 rpt 1	0.99	0.91	7.98E-08	0.0234	104000	2.25E-07	0.998	1.592
H3 rpt 2	1	0.72	6.25E-08	0.0195	118000	1.65E-07	0.996	1.893
H3 rpt 3	1	0.82	6.44E-08	0.0189	113000	1.68E-07	0.996	2.253
H3 rpt 4	1	1.05	8.46E-08	0.0149	84000	1.77E-07	0.998	1.645
H3 rpt 5	1	1.05	7.92E-08	0.0149	83000	1.79E-07	0.997	2.048
**H3 average**			**7.41E-08**	**0.01832**	**100400**	**1.83E-07**		

Supplementary Table 2: Crystallographic data collection, refinement and validation statistics.

	ChuA-heme	Binder C8
**Data Collection**[a]		
Space Group	$P2_12_12_1$	$P2_12_12_1$
Cell Dimensions		
a, b, c (Å)	78.21, 116.12, 123.34	85.37, 110,30, 127.34
α, β, γ (°)	90, 90, 90	90, 90, 90
Wavelength	0.987	0.987
Resolution (Å)	48.43-2.80 (2.95-2.80)	46.32-2.46 (2.55-2.46)
R_{merge}	0.189 (3.760)	0.179 (1.478)
R_{pim}	0.090 (1.790)	0.113 (0.946)
I/s(*I*)	8.9 (0.8)	5.7 (0.8)
CC(1/2)	0.999 (0.442)	0.997 (0.648)
Completeness (%)	99.9 (99.8)	99.4 (95.0)
Redundancy	10.0 (9.8)	6.7 (6.4)
No. reflection	28374 (4040)	44074 (4383)
**Refinement statistics**		
R_{work}/R_{free}	0.253/0.299	0.302/0.324
No. atoms		
Protein	4782	7525
Ligand / ions	42	0
Solvent	n/a	0
R.m.s deviations		
Bond lengths (Å)	0.003	0.007
Bond angles (°)	0.647	1.198
Ramachandran Plot		
Favored/Allowed/Outliers (%)	94.89/4/94/0.16	97.36/2.17/0
Clash Score	11.09	13.45
Average B-factor	104.19	63.83
Molprobity Score	1.91	1.94
PDB ID	9DHE	9DIV

[a] Values in parentheses are for highest-resolution shell.

Data from one crystal was collected for each structure

Supplementary Table 3: Comparison of RMSD of C8 molecules in the crystal and RFdiffusion model

		C8 AF2	C8 cryst #1	C8 cryst #2	C8 cryst #3	C8 cryst #4	C8 cryst #5	C8 cryst #6	C8 cryst #7	C8 cryst #8
**Molecule R.M.S.D**	**C8 AF2**	0	0.658	0.9	0.636	0.525	0.703	0.79	0.528	0.55
**Atoms Aligned**	**C8 AF2**	999	658	721	759	737	617	733	758	721

Supplementary Table 4: Cryo-EM data collection, refinement and validation statistics. All datasets were collected with a zero-loss filtering slit width of 10 eV and with 70 frames per movie.

	ChuA-G7 (EMD-46916) (PDB 9DIR)	ChuA-H3 (EMD-46917) (PDB 9DIS)
**Data collection and processing**		
Magnification	105kx	105kx
Voltage (kV)	300	300
Electron exposure (e–/Å^2)	70.0	70.0
Defocus range (μm)	-1.4 -0.5	-1.4 -0.5
Pixel size (Å)	0.82	0.82
Symmetry imposed	C1	C1
Initial particle images (no.)	3,850,705	2,490,792
Final particle images (no.)	131,924	333,575
Map resolution (Å)	2.97	2.51
FSC threshold	0.143	0.143
Map resolution range (Å)	2.63 – 4.72	2.23 – 4.62
**Refinement**		
Initial model used (PDB code)	3FHH	3FHH
Model resolution (Å)	2.97	2.51
FSC threshold	0.143	0.143
Model resolution range (Å)	n/a	n/a
Map sharpening *B* factor (Å^2)	85.6	82.1
Map-Model CC	0.78	0.82
Model composition		
Non-hydrogen atoms	5870	5713
Protein residues	755	741
Ligands	0	0
Solvent	0	0
B factors (Å^2)		
Protein	108.92	45.43
Ligand	n/a	n/a
Solvent	n/a	n/a
R.m.s. deviations		
Bond lengths (Å)	0.003	0.004
Bond angles (°)	0.595	0.541
Validation		
MolProbity score	1.76	1.71
Clashscore	11.94	10.92
Poor rotamers (%)	0.00	0.00
Ramachandran plot		
Favored (%)	97.05	97.41
Allowed (%)	2.95	2.86
Disallowed (%)	0.00	0.00
