# Supplementary material for: Inhibiting heme piracy by pathogenic Escherichia coli using de novo-designed proteins
Source: Nat Commun. 2025 Jul 9;16:6066. doi: 10.1038/s41467-025-60612-9 (PMC12241658; doi:10.1038/s41467-025-60612-9)
Supplement: Supplementary file 2 — Description of Additional Supplementary Files [file 41467_2025_60612_MOESM2_ESM.pdf]

Description of Additional Supplementary Data (DOASF):

Supplementary Data 1. AlphaFold3 models of wildtype and heme-coordinating histidine mutants in complex with heme.

Supplementary Data 2. Nucleotide and amino acid sequences of the four top ChuA binders. The sequence includes a 6xhistidine affinity tag used for purification.

Supplementary Data 3. AlphaFold2 models of ChuA in complex with the four top ChuA binders.

Supplementary Data 4. Oligonucleotides and synthetic DNA constructs used in this study
